# Effect of some prostanoid receptor modulators on cholinergic and purinergic pathways in rat model of hemorrhagic cystitis: potential beneficial action of selexipag and alprostadil

**DOI:** 10.1007/s00210-025-04632-8

**Published:** 2025-11-13

**Authors:** Evelyn M. Gerges, Mai M. Helmy, Tahia T. Daabees, Amira M. Senbel

**Affiliations:** 1 Department of Pharmacology and Toxicology, Faculty of Pharmacy, Alexandria University, Alazarita, 21521 Alexandria Egypt; 2 Biological and Clinical Sciences Division, College of Pharmacy, Arab Academy for Science, Technology and Maritime Transport, Alexandria, Egypt; 3 College of Pharmacy, Al-Farahidi University, Baghdad, 10021, Iraq

**Keywords:** Hemorrhagic cystitis, Prostanoid, Detrusor muscle, Selexipag, Alprostadil

## Abstract

Acetylcholine (ACh) and adenosine triphosphate (ATP) are the major neurotransmitters in bladder contraction. This study investigates the modulatory role of some prostanoids in cholinergic and purinergic pathways in normal and cystitis. Cystitis was induced in rats by cyclophosphamide single injection (300 mg/kg) and confirmed histopathologically. Organ bath experiments were implemented using isolated detrusor muscles. Bladder contraction was induced by electrical stimulation and by ACh or ATP direct addition. Cystitis attenuated bladder contractility. Alprostadil and selexipag amplified neurogenic and cholinergic contractions in normal and inflamed bladder. The neurogenic contractions amplification was higher in cystitis with 54.63 ± 42.11% compared to 18.77 ± 5.98% in normal at 10^−7^ M selexipag. While cholinergic contraction amplification was higher in cystitis using selexipag and alprostadil, the amplifying effect of 10^−6^ M alprostadil was 21.07 ± 15.07% and 128.02 ± 76.61% and the amplifying effect of 10^−6^ M selexipag was 39.51 ± 19.72% and 227.6 ± 229.59% in normal and cystitis respectively. At higher concentrations, selexipag and alprostadil amplified purinergic contractions in normal bladder. RO1138452 inhibited neurogenic and cholinergic contractions in normal and inflamed bladder, with less inhibition of cholinergic contractions in inflamed bladder with 26.6 ± 20.72% compared to 54.22 ± 19.6% in normal. The same was observed using SC51322 but cholinergic contraction inhibition was not significant in normal. Both antagonists inhibited purinergic contractions in normal. S18886 inhibited purinergic contractions in normal bladder. EP_1_ and IP receptors may play a role in bladder contraction. TP receptor is involved in purinergic signaling in normal physiology. Selexipag and alprostadil may have a potential beneficial action in cystitis and merit further investigation.

## Introduction

The bladder is an elastic, spherical organ located in the pelvic cavity. It is composed of two parts: the base, which includes the bladder neck and a triangular-shaped area known as the trigone, and the body, which is located above the ureter orifices (de Groat and Yoshimura 2015; Maynard and Downes 2019).

Urinary bladder disorders have a significant influence on social life and are thought to be among the most prevalent lower urinary tract disorders. Overactive bladder (OAB), inflammatory diseases such hemorrhagic cystitis, obstruction of the bladder outlet, and bladder cancer are examples of urinary bladder disorders (Andersson 2016). Hemorrhagic cystitis is a pathological condition in which there is misery from the urinary bladder irritation accompanied by hematuria (Gorczynska et al. 2005) in absence of any other condition like vaginal bleeding, hypocoagulability, or sepsis (El-Zimaity et al. 2004). This condition is accompanied by symptoms such as dysuria, urgency, frequency, and nocturia. Chemotherapeutic agents, remarkably cyclophosphamide (CYP), can be considered a common cause of this condition (Stillwell and Benson 1988). It has been reported that 25% of patients suffer from hemorrhagic cystitis when they use high doses of CYP (Zhang et al. 2021). Hemorrhagic cystitis can be lethal with a reported mortality rate of 9.1% (Kaplan and Wolf 2009).

Acetylcholine (ACh) and adenosine triphosphate (ATP) are the two main neurotransmitters that affect the contractility of the bladder detrusor smooth muscle (de Groat and Yoshimura 2015; Patel and Chapple 2008). Muscarinic receptor subtypes M_2_ and M_3_ are the dominant subtypes in bladder detrusor muscle. The M_3_ subtype, despite being less abundant than the M_2_ subtype, is more involved in bladder contractility (Hegde and Eglen 1999; Matsui et al. 2002; Wang et al. 1995). The M_3_ receptor belongs to the G_q/11_ family, resulting in the stimulation of phospholipase C (PLC) and inositol triphosphate (IP_3_) and the release of intracellular calcium (Ca^2+^). The M_2_ receptor belongs to the G_i/o_ family, inhibiting the activity of adenylyl cyclase (AC) (Andersson and Arner 2004). ATP activates the purinergic P2X receptors in bladder detrusor muscles. P2X family receptors are ligand-gated ionotropic channels that, upon activation, increase the extracellular Ca^2+^ influx, resulting in bladder contraction (Andersson and Arner 2004; Burnstock and Williams 2000). The P2X family includes seven subtypes, and immunohistochemical experiment in rat showed that P2X_1_ is the prevailing subtype in the detrusor muscle membrane (Lee et al. 2000) and ATP-induced contractions were abolished in mice lacking the P2X_1_ receptor (Vial and Evans 2000).

Prostanoids, prostaglandins (PGs) and thromboxanes, are signaling molecules produced under normal and pathological conditions and synthesized by cyclooxygenase (COX) enzyme. PGE_2_, prostacyclin (PGI_2_), PGD_2_, PGF_2α_, and thromboxane A_2_ (TXA_2_) are the main prostanoids produced in the bladder. These substances act through prostanoid receptors, which are EP_1-4_, IP, DP, FP, and TP, respectively (Rahnama'i et al. 2012). Jeremy et al. (1984) reported that in rat urinary bladder, the major type of prostanoids is PGI_2_ followed by PGE_2_ and TXA_2_, whereas, in human urinary bladder mucosa, the major type of prostanoids is PGI_2_ followed by PGE_2_, PGF_2α_, and TXA_2_ (Jeremy et al. 1987). It has been observed that EP_1_ and EP_3_ receptors play a role in bladder contraction, whereas EP_2_ and EP_4_ receptors play a role in bladder relaxation (Coleman et al. 1994; Hata and Breyer 2004). PGE_2_ increases the activity of isolated bladder smooth muscles in mice (Kobayter et al. 2012) and induces the contraction of isolated rat bladder smooth muscles, an effect that was inhibited in the presence of either EP_1_ or EP_3_ antagonist (Root et al. 2015). It has been debatable how prostanoids interact with the cholinergic and purinergic pathways, which are the two main pathways controlling the bladder contractility. In this regard, the current study aims to investigate this interaction using some prostanoid modulators in a rat model of CYP-induced hemorrhagic cystitis in comparison to normal rats to examine their possible clinical significance in hemorrhagic cystitis.

## Materials and methods

### Animals

Experiments were performed using male Wistar rats weighing (180–280 g) obtained from the animal house of Faculty of Pharmacy, Alexandria University. Rats were kept at room temperature with free access to chow (19% proteins, Elfagr Co., Egypt) and water. The laboratory animal care principles were strictly followed in all research experimental protocols and procedures as approved by the Institutional Animal Care and Use Committee of the Faculty of Pharmacy, Alexandria University (Approval Number: AU06.2020.1.12.1.67).

No animals were excluded. In addition, animals were randomly chosen for the induction of hemorrhagic cystitis model and for in vitro organ bath experiments.

### Chemicals

The chemicals utilized in the current study and their respective sources are represented as follows: acetylcholine chloride (ACh, sigma), adenosine 5′-triphosphate dipotassium salt dihydrate (ATP, sigma), alprostadil (Alfaprostin® ampoule, Rotabiogen Pharm. Co.), cyclophosphamide (CYP, Endoxan® vial, Baxter), 4,5-dihydro-N-[4-[[4-(1-methylethoxy)phenyl]methyl]phenyl]−1H-imidazol-2-amine hydrochloride (RO1138452 hydrochloride, Tocris), (6*R*)−6-[[(4-chlorophenyl)sulfonyl]amino]−5,6,7,8-tetrahydro-2-methyl-1-naphthalenepropanoic acid (S18886, Tocris), 8-chloro-2-[3-[(2-furanylmethyl)thio]−1-oxopropyl]-dibenz(Z)[b,f][1,4]oxazepine-10(11H)-carboxylic acid hydrazide (SC51322, Tocris), selexipag (Tocris), and thiopental sodium (Thiopental sodium® vial, EIPICO). The solutions of ACh and ATP were prepared in distilled water. The stock solutions of alprostadil, RO1138452 hydrochloride, S18886, SC51322, and Selexipag were prepared in 95% ethanol and serially diluted with physiological saline. Thiopental sodium and CYP were dissolved in physiological saline.

### Induction of hemorrhagic cystitis

Each rat received a single intraperitoneal (I.P.) dose of CYP (300 mg/kg) (Wada et al. 2013). After 48 h, the rats were anesthetized by I.P. injection of thiopental (50 mg/kg) to isolate the urinary bladders and then the animals were sacrificed by exsanguination. Some of the urinary bladders were kept in formalin and prepared for histopathological examination and the others were used for in vitro tension study experiments.

### Histopathological examination of rat urinary bladder

Histopathology was performed to confirm the induction of hemorrhagic cystitis after treatment with CYP. The isolated urinary bladders were kept for 24 h in 10% formalin. After that, the isolated tissues were embedded in paraffin and stained with hematoxylin–eosin (H & E).

### Preparation of isolated rat urinary bladder smooth muscle

The urinary bladder was isolated and the detrusor smooth muscle was prepared as described by Luheshi and Zar (1991). The lower abdomen was opened and the bladder was isolated and any blood vessels or connective tissues were removed from the outer surface of the bladder. After that, any residual urine was removed from the bladder and the bladder was washed several times using Krebs solution and fixed from its apex in a petri dish containing Krebs solution. The bladder was unfolded to give a rectangular sheet that was cut longitudinally to give two bladder detrusor muscle strips 2 × 15 mm. The detrusor muscle was fixed at one end between two parallel platinum electrodes 4–5 mm apart and mounted in 25-mL organ bath containing Krebs solution (composition in mM; NaCl 118, KCl 4.7, MgSO_4_·7H_2_O 1.2, KH_2_PO_4_·1.2, NaHCO_3_ 25, glucose monohydrate 11 and CaCl_2_·2H_2_O 2.5), kept at 37 °C and continuously aerated with carbogen (95% O_2_ and 5% CO_2_). The other end was tied and attached to a force displacement transducer (Grass FT-03) which in turn was connected to a computerized data acquisition system through an MLAC11 Grass adapter cable. Lab Chart-7 pro software (Power Lab 4/35, model ML 866/P; AD Instrument Pty Ltd., Castle Hill, Australia) was used to record the readings from tension study experiments. The tissue was left under a resting tension of 0.5 g to equilibrate for 30 min and during this period, the Krebs solution was replaced each 15 min.

For electrical field stimulation (EFS) experiments, two parallel platinum electrodes were used and they were attached to a Grass electronic stimulator (Model S 48). Each detrusor muscle strip was either exposed to electric stimulation in increasing frequencies (1, 4, and 16 Hz; voltage 80 V and 1-ms pulse duration) (Parija et al. 1991) or exposed to submaximal frequency of 4 Hz. After the incubation period of the tested drug, each strip was re-exposed to either frequency response curve or submaximal frequency of 4 Hz. The contractile response was measured before and after the addition of the tested drug and the percentage change compared to the basal value before the drug addition was calculated.

For ACh and ATP-induced contraction experiments, each detrusor muscle strip was either exposed to a cumulative concentration response curve (CRC) of ACh (10^−9^–10^−3^ M) or ATP (10^−9^–10^−3^ M) or exposed to submaximal concentration of ACh (10^−4^ M) or ATP (10^−4^ M) and then the preparation was washed several times using Krebs solution before the addition of tested drug. After the incubation period of the tested drug, each strip was re-exposed to either cumulative CRC or submaximal concentration (10^−4^ M) of ACh or ATP. The contractile response was measured before and after the addition of the tested drug and the percentage change compared to the basal value before the drug addition was calculated.

### Experimental protocols

#### The effect of CYP-induced hemorrhagic cystitis on EFS, ACh, and ATP-induced contraction of isolated rat detrusor muscle

The effect of CYP-induced hemorrhagic cystitis on EFS using frequency response curve (1–16 Hz), ACh (10^−9^–10^−3^ M) and ATP (10^−9^–10^−3^ M)-induced contraction of isolated rat detrusor muscles was compared to normal rats. ACh and ATP were added as a cumulative CRC.

#### The effect of some prostanoid receptor agonists on EFS, ACh, and ATP-induced contraction of isolated detrusor muscle in normal rats and rats with CYP-induced hemorrhagic cystitis

The effect of non-cumulative concentrations of alprostadil (synthetic PGE_1_, 10^−9^–10^−6^ M) and selexipag (selective IP receptor agonist, 10^−9^–10^−6^ M) was tested, after incubation period of 10 min, on the contractility of isolated rat detrusor muscle induced by submaximal frequency of (4 Hz), submaximal ACh concentration (10^−4^ M) and submaximal ATP concentration (10^−4^ M) in normal rats and rats with CYP-induced hemorrhagic cystitis.

#### The effect of some prostanoid receptor antagonists on EFS, ACh, and ATP-induced contraction of isolated detrusor muscle in normal rats and rats with CYP-induced hemorrhagic cystitis

The effect of SC51322 (potent EP_1_ receptor antagonist), RO1138452 hydrochloride (selective IP receptor antagonist), and S18886 (potent TXA_2_ (TP) antagonist) was tested, after incubation period of 30 min, on the contractility of isolated rat detrusor muscle induced by frequency response curve (1–16 Hz), cumulative CRC of ACh (10^−9^–10^−3^ M) and ATP (10^−9^–10^−3^ M) in normal rats and rats with CYP-induced hemorrhagic cystitis.

### Data analysis and statistics

Values were expressed as mean ± SD. Student *t*-test was used for the comparison between two groups. For multiple comparisons, analysis of variance (ANOVA) was used followed by Bonferroni’s post hoc test, for verifying statistical significance. These analyses were performed using SigmaPlot 14 and graphs were represented using GraphPad Prism 8. Probability levels, as a descriptive value, less than 0.05 were considered significant.

## Results

### Histopathological examination of rat urinary bladder

**Fig. 1:**
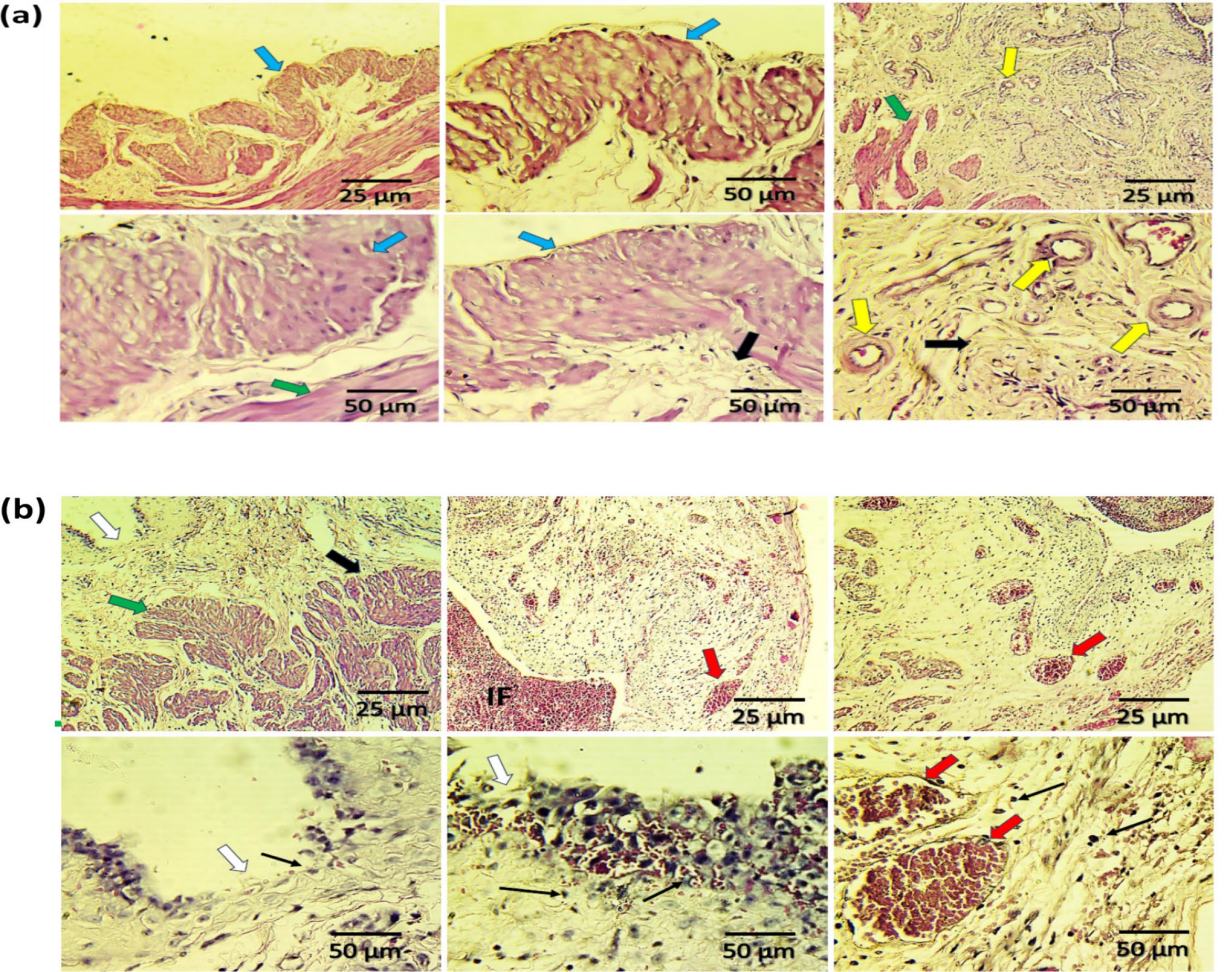
Hematoxylin and eosin (H and E) stained photomicrographs (magnification 100 × and 400 ×) of urinary bladders obtained from control (**a**) and cyclophosphamide (CYP, 300 mg/kg)-treated rats (**b**). The normal urinary bladder wall was composed of the urothelium formed by firmly stuffed transitional cells showing the superficial umbrella cells with minimal intercellular space (light blue arrows). The connective tissues (thick black arrows) which separate the epithelium from the underlying lamina propria (green arrows) were intact and may vary in thickness, showing normal blood vessels (yellow arrows) with minimal infiltration of immune cells (**a**). White arrows point to severe damage with desquamation of the surface epithelium. Thin black arrows point to marked swelling of the epithelial cell and inflammatory cell infiltration in the mucosal and the sub-urothelial connective tissues of the urinary bladder. Marked interstitial fibrosis was also noted in lamina propria (IF) with marked damage and subsequent bleeding (red arrows) (**b**). The scale bar of 25 μm corresponds to magnification power 100 ×, while that of 50 μm corresponds to magnification power 400 ×

As shown in Fig. 1b, compared to control rats, cyclophosphamide administration resulted in cystitis manifested as severe damage with desquamation of the surface epithelium with marked infiltration of inflammatory cells and showing hemorrhagic features. Furthermore, marked edema in the lamina propria and cloudy swelling of the epithelial cells were observed, as well as inflammatory cell infiltration in the mucosal and the sub-urothelial connective tissues of the urinary bladder, leading to vascular congestion, swelling and damage, and subsequent bleeding. Marked Interstitial fibrosis was also noted in lamina propria.

#### The effect of CYP-induced hemorrhagic cystitis on EFS, ACh, and ATP-induced contraction of isolated rat detrusor muscle

**Fig. 2:**
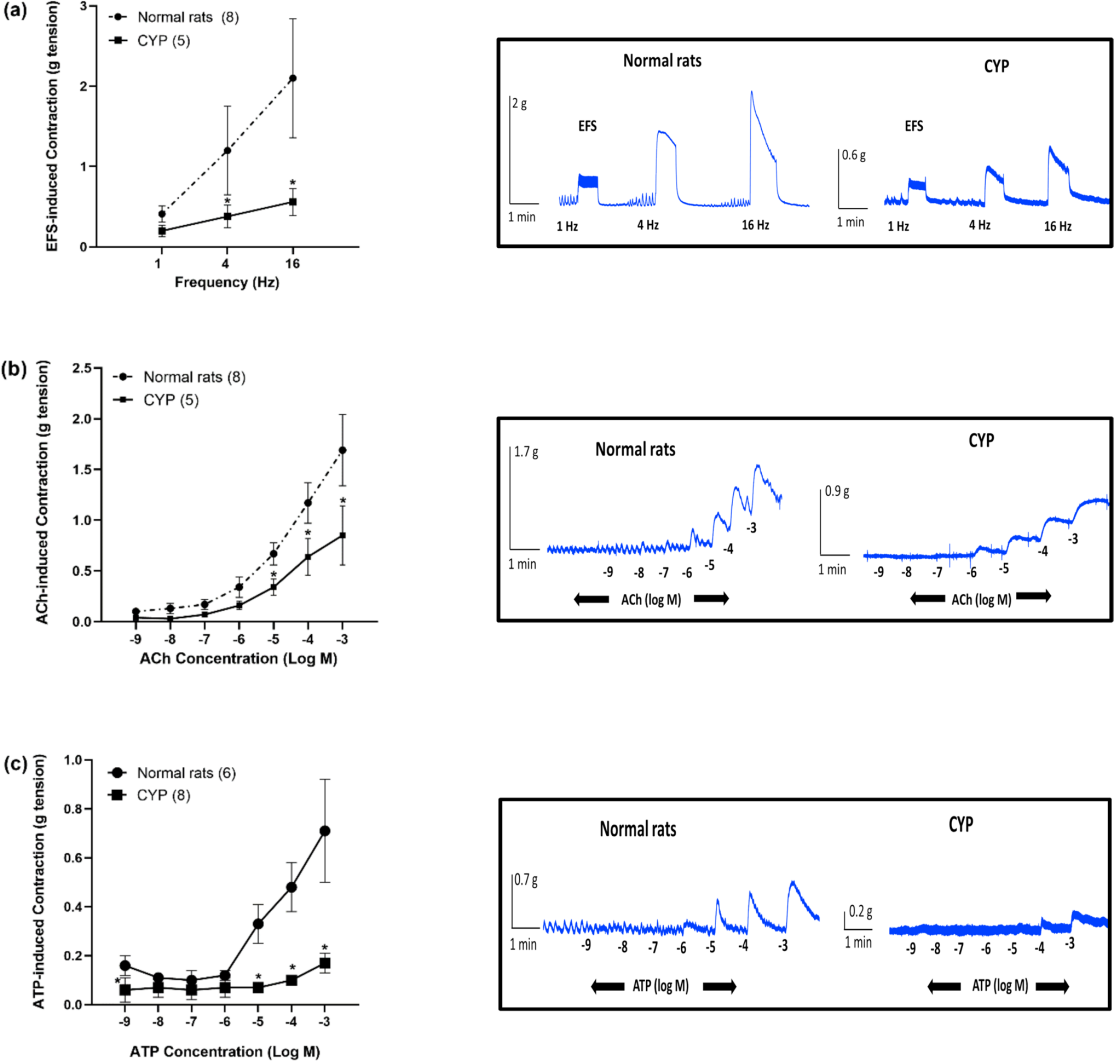
Effect of cyclophosphamide (CYP, 300 mg/kg) injection on EFS (1–16 Hz, **a**), ACh (10^−9^–10^−3^ M, **b**), and ATP (10^−9^–10^−3^ M, **c**)-induced contraction of isolated rat detrusor muscles. Responses are expressed as mean ± SD. * denotes significant difference from normal rats (*p* < 0.05). Values between parentheses indicate the number of animals

In normal rats, EFS (1–16 Hz) induced frequency-dependent contraction of the isolated rat detrusor muscle with a contraction of 2.10 ± 0.74 g (at 16 Hz). The cumulative addition of ACh (10^−9^–10^−3^ M) induced concentration-dependent contraction with a contraction of 1.69 ± 0.35 g (at 10^−3^ M). The cumulative addition of ATP (10^−9^–10^−3^ M) induced contraction of 0.16 ± 0.04 g (at 10^−9^ M), then there was a slight decrease in contraction (at 10^−8^ M) followed by concentration-dependent contraction with a contraction of 0.71 ± 0.21 g (at 10^−3^ M). CYP caused a significant inhibition of EFS-induced contraction (at 4 Hz) and (at 16 Hz) with a contraction of 0.38 ± 0.14 g and 0.56 ± 0.17 g respectively. CYP also caused a significant inhibition of ACh-induced contraction at concentrations (10^−5^–10^−3^ M) with a contraction of 0.85 ± 0.29 g (at 10^−3^ M). ATP-induced contraction was significantly reduced (at 10^−9^ M) and at concentrations (10^−5^–10^−3^ M) with a contraction of 0.17 ± 0.04 g (at 10^−3^ M) (Fig. 2).

#### The effect of some prostanoid receptor agonists on EFS, ACh, and ATP-induced contraction of isolated detrusor muscle in normal rats and rats with CYP-induced hemorrhagic cystitis

**Fig. 3:**
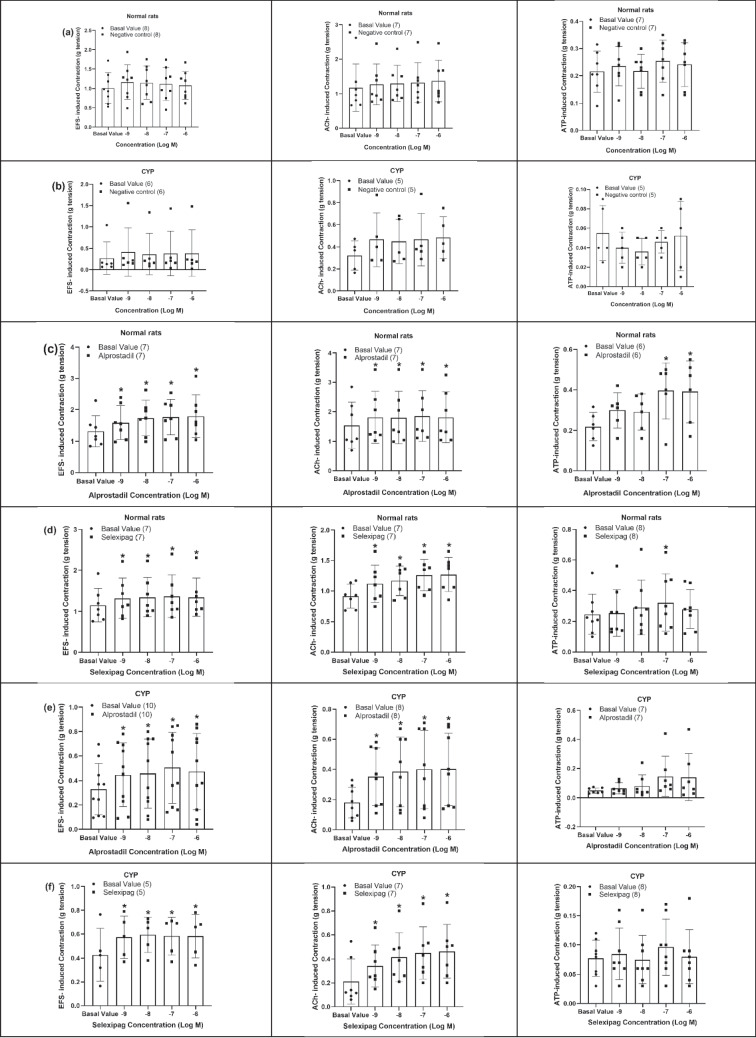
Effect of negative control experiment of prostanoid receptor agonists (10^**−**9^–10^**−**6^ M) on EFS (4 Hz), ACh (10^**−**4^ M), and ATP (10^**−**4^ M)-induced contraction of isolated detrusor muscles in normal rats (**a**) and rats with CYP-induced hemorrhagic cystitis (**b**). Effect of alprostadil (synthetic PGE_1_, 10^**−**9^–10^**−**6^ M) on EFS (4 Hz), ACh (10^**−**4^ M), and ATP (10^**−**4^ M)-induced contraction of isolated detrusor muscles in normal rats (**c**) and rats with CYP-induced hemorrhagic cystitis (**e**). Effect of selexipag (selective IP receptor agonist, 10^**−**9^–10^**−**6^ M) on EFS (4 Hz), ACh (10^**−**4^ M), and ATP (10^**−**4^ M)-induced contraction of isolated detrusor muscles in normal rats (**d**) and rats with CYP-induced hemorrhagic cystitis (**f**). Responses are expressed as mean ± SD. * denotes significant difference from the basal value before the drug addiction (*p* < 0.05). Values between parentheses indicate the number of animals

The negative control experiments (using the vehicle, 95% ethanol serially diluted with physiological saline, only without the drug) were conducted on both normal and CYP-injected rats and there was no significant change in EFS (4 Hz), ACh (10^−4^ M) or ATP (10^−4^ M)-induced contraction (Fig. 3a, b).

In normal rats, alprostadil significantly amplified both EFS (4 Hz) and ACh (10^−4^ M)-induced contraction at concentrations (10^−9^–10^−6^ M). The EFS-induced contraction was amplified from 1.32 ± 0.46 g to 1.8 ± 0.62 g and ACh-induced contraction was amplified from 1.54 ± 0.73 g to 1.81 ± 0.79 g (at 10^−6^ M) of alprostadil. Regarding ATP (10^−4^ M)-induced contraction, alprostadil also caused a significant amplification in the contractility but at high concentrations only (10^−7^–10^−6^ M) from 0.22 ± 0.06 g to 0.39 ± 0.14 g (at 10^−6^ M) (Fig. 3c). A significant amplification of both EFS (4 Hz) and ACh (10^−4^ M)-induced contraction was also observed in case of selexipag (10^−9^–10^−6^ M). EFS and ACh-induced contraction was amplified from 1.15 ± 0.38 g to 1.35 ± 0.43 g and from 0.92 ± 0.18 g to 1.27 ± 0.26 g respectively (at 10^−6^ M) of selexipag. Regarding ATP (10^−4^ M)-induced contraction, the amplification was significant only at concentration (10^−7^ M) of selexipag where contraction was amplified from 0.25 ± 0.12 g to 0.32 ± 0.17 g (Fig. 3d).

**Fig. 4:**
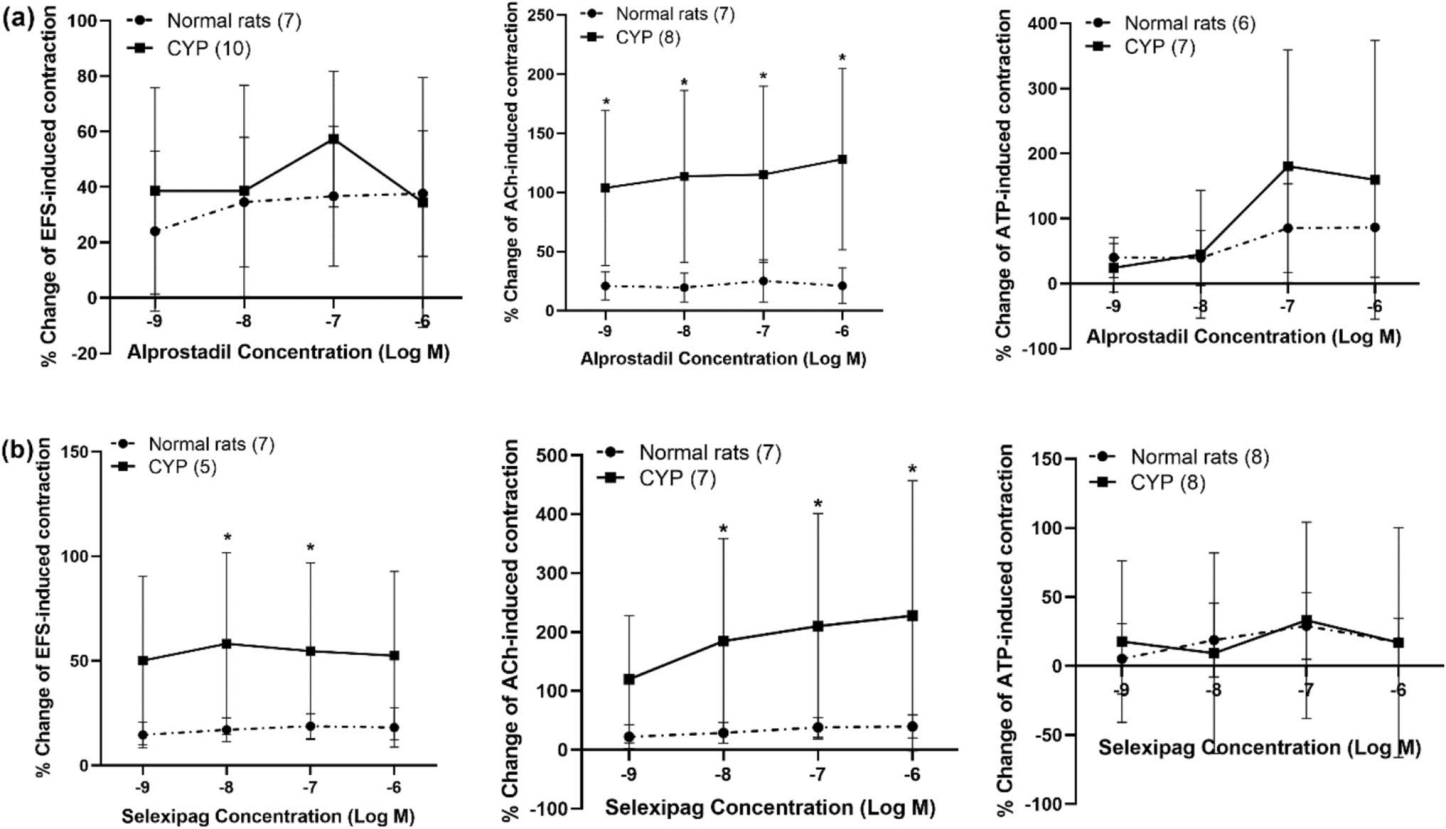
Percentage change induced by alprostadil (synthetic PGE_1_, 10^**−**9^–10^**−**6^ M, **a**) and selexipag (selective IP receptor agonist, 10^**−**9^–10^**−**6^ M, **b**) on EFS (4 Hz), ACh (10^**−**4^ M), and ATP (10^**−**4^ M)-induced contraction of isolated detrusor muscles in normal rats and rats with CYP-induced hemorrhagic cystitis. Responses are expressed as mean ± SD. * denotes significant difference from normal rats (*p* < 0.05). Values between parentheses indicate the number of animals

In rats with CYP-induced hemorrhagic cystitis, both alprostadil and selexipag (10^−9^–10^−6^ M) caused a significant amplification of EFS (4 Hz) and ACh (10^−4^ M)-induced contraction. In case of alprostadil, EFS and ACh-induced contraction was amplified from 0.33 ± 0.2 g to 0.47 ± 0.3 g and from 0.18 ± 0.1 g to 0.4 ± 0.22 g respectively (at 10^−6^ M). In the case of selexipag (10^−6^ M), EFS (4 Hz) and ACh (10^−4^ M)-induced contraction was amplified from 0.43 ± 0.2 g to 0.58 ± 0.16 g and from 0.21 ± 0.17 g to 0.46 ± 0.21 g respectively. When compared with normal rats, it was observed that amplification in EFS-induced contraction in case of cystitis was significantly higher in the presence of selexipag (10^−8^–10^−7^ M). The amplification attained in cystitis was 54.63 ± 42.11% compared to 18.77 ± 5.98% in normal rats (at 10^−7^ M). In the case of ACh-induced contraction, the amplification induced by alprostadil in rats with cystitis was significantly higher compared to normal rats at concentrations (10^−9^–10^−6^ M) and the amplification induced by selexipag in rats with cystitis was also significantly higher compared to normal rats at concentrations (10^−8^–10^−6^ M). The amplifying effect of alprostadil (at 10^−6^ M) on ACh-induced contraction was 21.07 ± 15.07% and 128.02 ± 76.61% in normal rats and CYP-injected rats respectively and the amplifying effect of selexipag (at 10^−6^ M) on ACh-induced contraction was 39.51 ± 19.72% and 227.6 ± 229.59% in normal rats and CYP-injected rats respectively. On the other hand, neither alprostadil nor selexipag made a significant change in ATP-induced contraction in CYP-injected rats (Figs. 3e, f and 4).

#### The effect of some prostanoid receptor antagonists on EFS, ACh, and ATP-induced contraction of isolated detrusor muscle in normal rats and rats with CYP-induced hemorrhagic cystitis

**Fig. 5:**
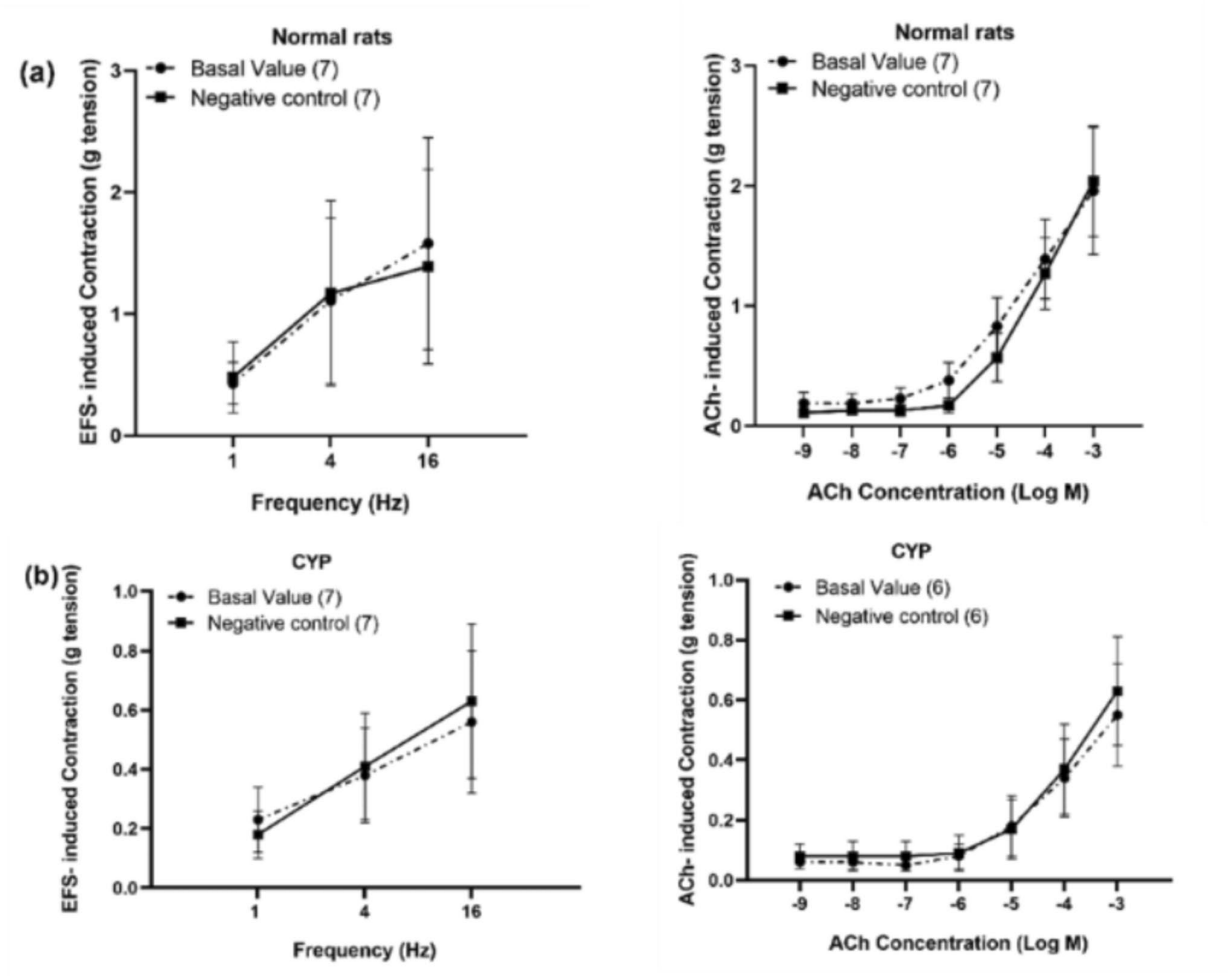

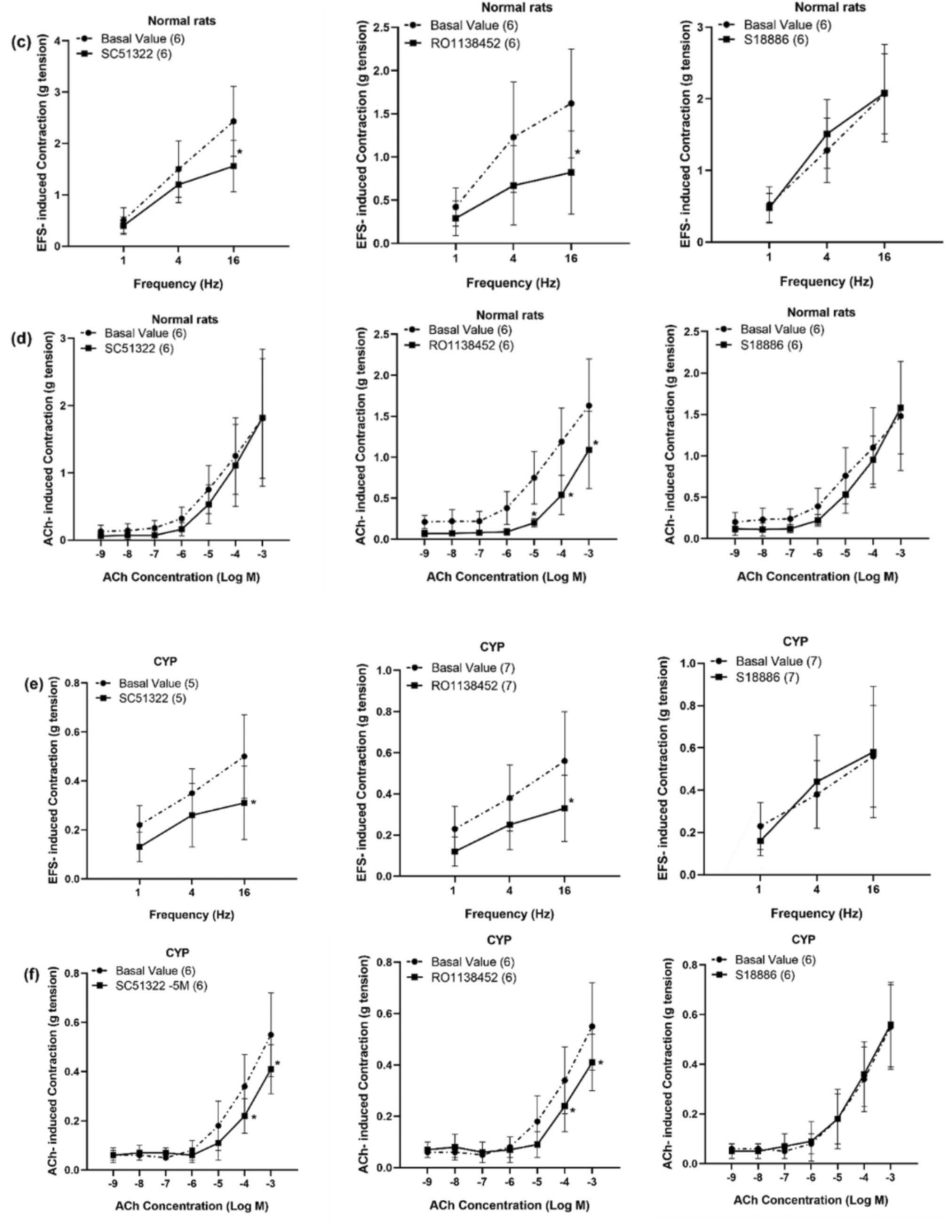
Effect of negative control experiment of prostanoid receptor antagonists (10^**−**5^ M) on EFS (1–16 Hz) and ACh (10^**−**9^–10^**−**3^ M)-induced contraction of isolated detrusor muscles in normal rats (**a**) and rats with CYP-induced hemorrhagic cystitis (**b**). Effect of SC51322 (EP_1_ receptor antagonist, 10^**−**5^ M), RO1138452 (selective IP receptor antagonist, 10^**−**5^ M) and S18886 (TP antagonist, 10^**−**5^ M) on EFS (1–16 Hz)-induced contraction of isolated detrusor muscles in normal rats (**c**) and rats with CYP-induced hemorrhagic cystitis (**e**). Effect of SC51322 (10^**−**5^ M), RO1138452 (10^**−**5^ M), and S18886 (10^**−**5^ M) on ACh (10^**−**9^–10^**−**3^ M)-induced contraction of isolated detrusor muscles in normal rats (**d**) and rats with CYP-induced hemorrhagic cystitis (**f**). Responses are expressed as mean ± SD. * denotes significant difference from the basal value before the drug addition (*p* < 0.05). Values between parentheses indicate the number of animals

The negative control experiments (using the vehicle, 95% ethanol serially diluted with physiological saline) were conducted on both normal and CYP-injected rats and there was no significant change in EFS and ACh-induced contraction (Fig. 5a, b).

**Fig. 6:**
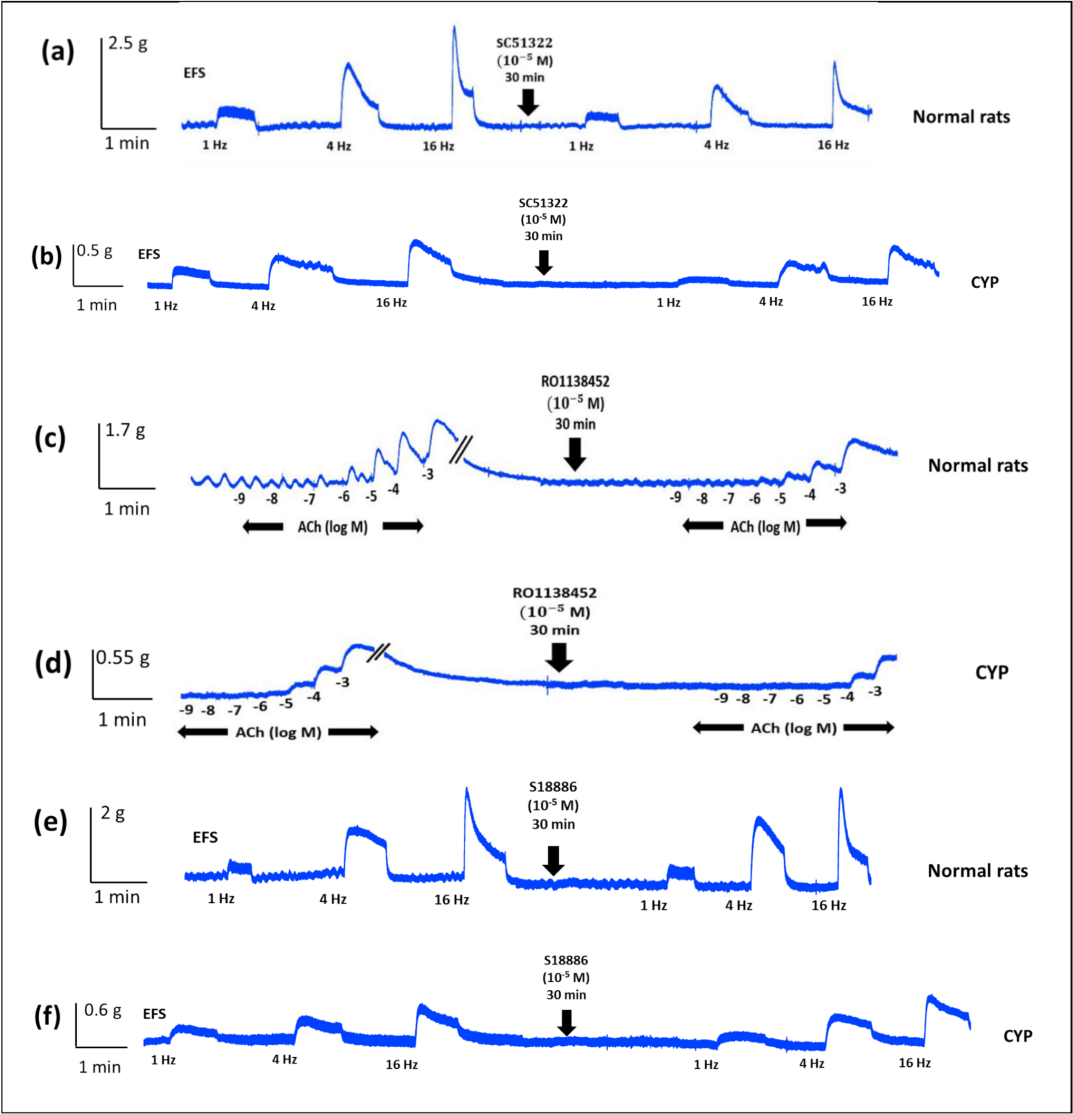
Representative tracings showing the effect of SC51322 (EP_1_ receptor antagonist, 10^**−**5^ M) on EFS (1–16 Hz)-induced contraction of isolated detrusor muscles in normal rats (**a**) and rats with CYP-induced hemorrhagic cystitis (**b**), the effect of RO1138452 (selective IP receptor antagonist, 10^**−**5^ M) on ACh (10^**−**9^–10^**−**3^ M)-induced contraction of isolated detrusor muscles in normal rats (**c**) and rats with CYP-induced hemorrhagic cystitis (**d**) and the effect of S18886 (TP antagonist, 10^**−**^^5^ M) on EFS (1–16 Hz)-induced contraction of isolated detrusor muscles in normal rats (**e**) and rats with CYP-induced hemorrhagic cystitis (**f**)

In normal rats, both SC51322 and RO1138452 (10^−5^ M) inhibited EFS-induced contraction and the inhibition was significant at frequency of (16 Hz) as the contraction decreased from 2.43 ± 0.68 g before the addition of SC51322 to 1.56 ± 0.5 g and from 1.62 ± 0.63 g before the addition of RO1138452 to 0.82 ± 0.48 g. ACh-induced contraction was also inhibited by both SC51322 and RO1138452 but the inhibition was only significant in case of RO1138452 and at high ACh concentrations (10^−5^–10^−3^ M). On the other hand, S18886 (10^−5^ M) had no significant effect on either EFS or ACh-induced contraction (Figs. 5c, d and 6a, c, e).

**Fig. 7:**
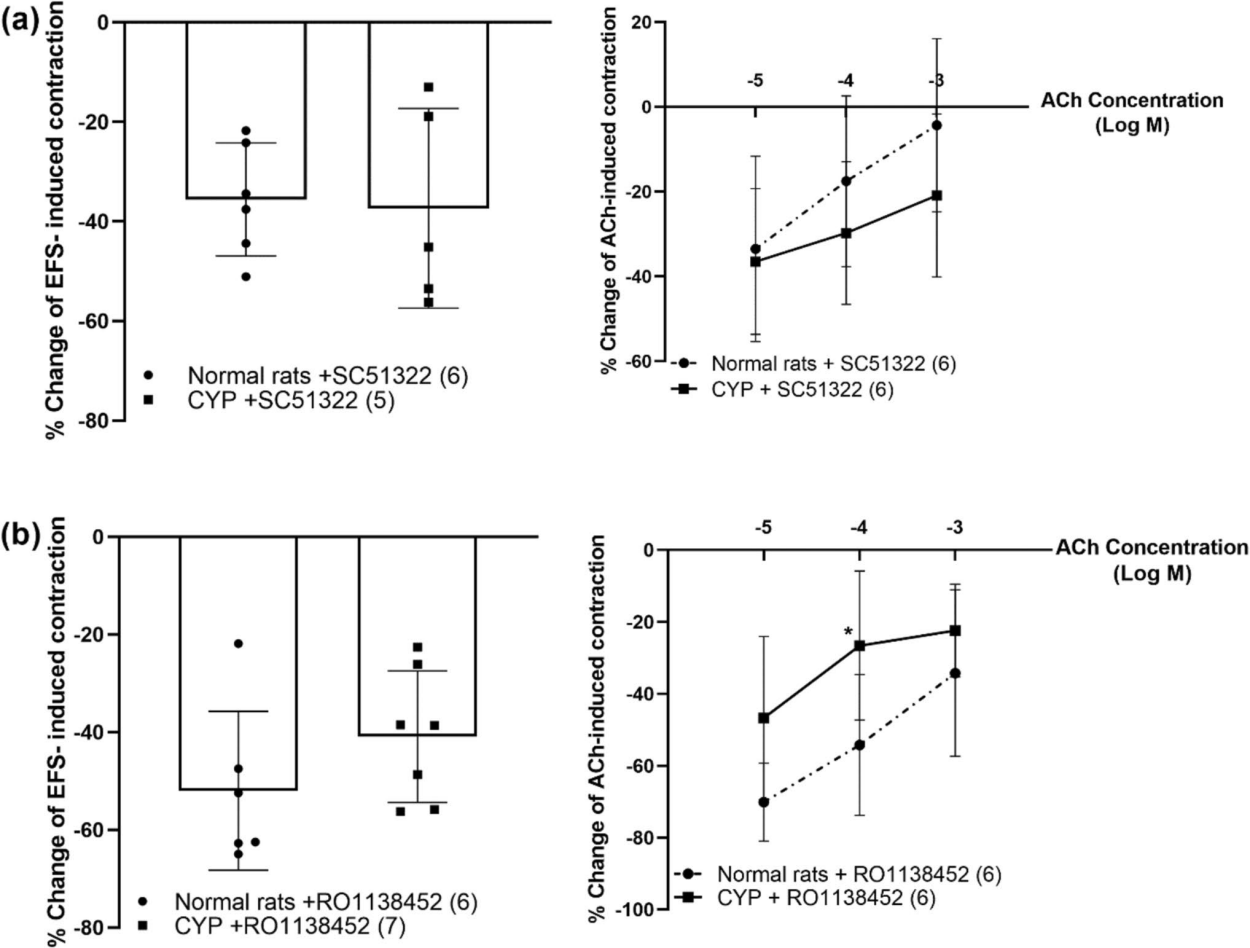
Percentage change induced by SC51322 (EP_1_ receptor antagonist, 10^−5^ M, **a**) and RO1138452 (selective IP receptor antagonist, 10^−5^ M, **b**) on EFS (16 Hz) and ACh (10^−5^–10^−3^ M)-induced contraction of isolated detrusor muscles in normal rats and rats with CYP-induced hemorrhagic cystitis. Responses are expressed as mean ± SD. * denotes significant difference from normal rats (*p* < 0.05). Values between parentheses indicate the number of animals

In CYP-injected rats, both SC51322 and RO1138452 produced a significant inhibition of EFS-induced contraction at frequency of (16 Hz) and ACh-induced contraction at concentrations (10^−4^–10^−3^ M). As in normal rats, S18886 had no significant change in either EFS or ACh-induced contraction (Figs. 5e, f and 6b, d, f). The inhibitory effect of SC51322 on EFS and ACh-induced contraction in CYP-injected rats was not significantly different compared to normal rats. In case of RO1138452, the inhibitory effect on EFS-induced contraction in CYP-injected rats was not significant from normal rats but the inhibitory effect on ACh (10^−4^ M)-induced contraction was significantly decreased from 54.22 ± 19.6% in normal rats to 26.6 ± 20.72% in CYP-injected rats (Fig. 7).

**Fig. 8:**
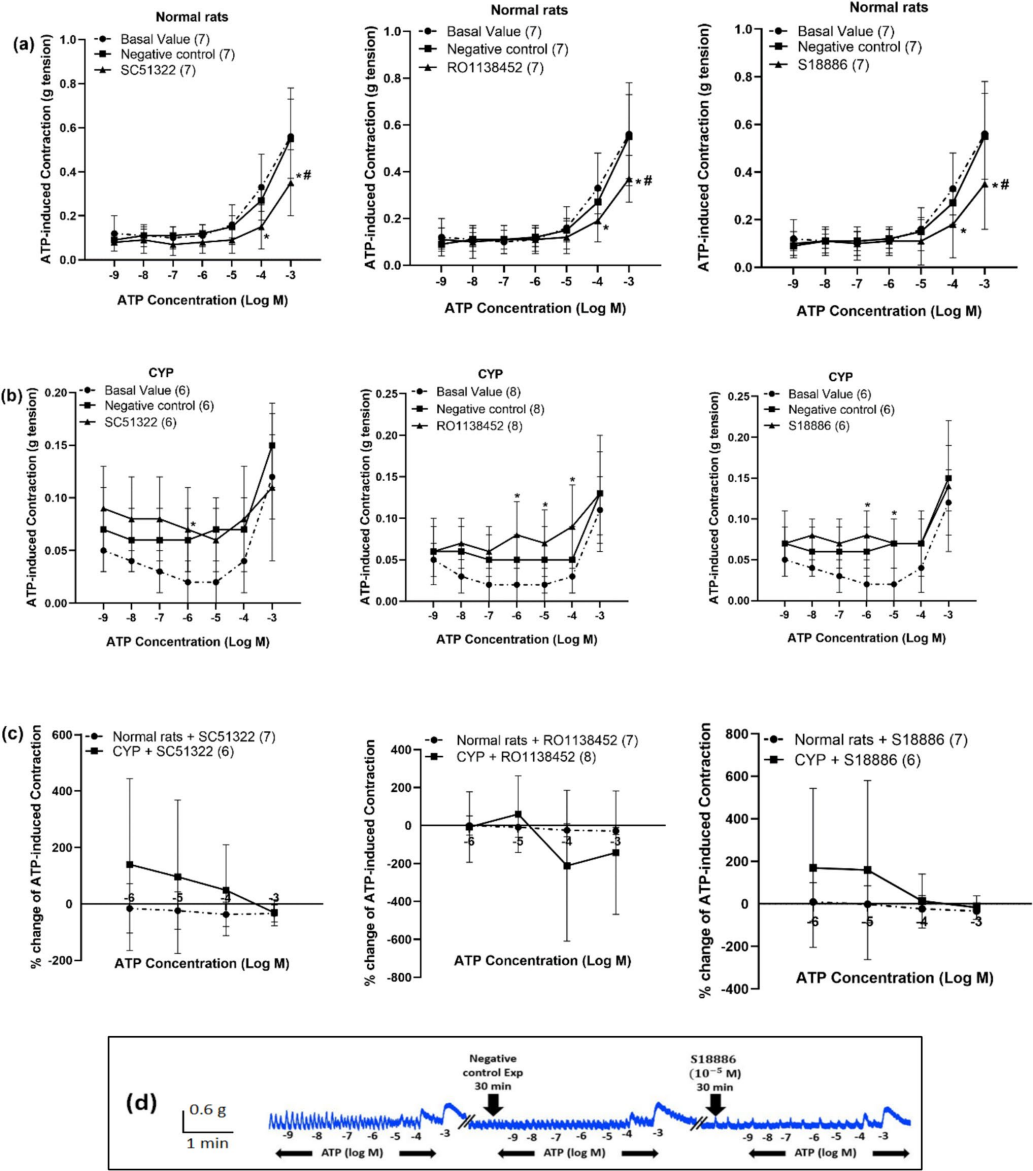
Effect of SC51322 (EP_1_ receptor antagonist, 10^−5^ M), RO1138452 (selective IP receptor antagonist, 10^−5^ M) and S18886 (TP antagonist, 10^−5^ M) on ATP (10^−9^–10^−3^ M)-induced contraction of isolated detrusor muscles in normal rats (panel, **a**) and rats with CYP-induced hemorrhagic cystitis (panel, **b**). Percentage change induced by SC51322 (10^−5^ M), RO1138452 (10^−5^ M) and S18886 (10^−5^ M) on ATP (10^−6^–10^−3^ M)-induced contraction of isolated detrusor muscles in normal rats and rats with CYP-induced hemorrhagic cystitis (**c**). Representative tracings showing the effect of S18886 (10^−5^ M, **d**) on ATP (10^−9^–10^−3^ M)-induced contraction of isolated detrusor muscles in normal rats. Responses are expressed as mean ± SD. * denotes significant difference from the basal value before the drug or its vehicle addition. # denotes significant difference from negative control experiment (*p* < 0.05). Values between parentheses indicate the number of animals

Regarding ATP-induced contraction, the effect of prostanoid receptor antagonists was compared to negative control experiment. In normal rats SC51322, RO1138452 and S18886 produced a significant inhibition of ATP-induced contraction compared to negative control experiment at ATP concentration (10^**−**3^ M). ATP (10^**−**3^ M)-induced contraction was decreased to 0.35 ± 0.15 g in the presence of SC51322 compared to 0.55 ± 0.18 g in negative control experiment and to 0.37 ± 0.1 g in presence of RO1138452 compared to 0.55 ± 0.18 g in negative control experiment, and in presence of S18886, it was decreased to 0.35 ± 0.19 g compared to 0.55 ± 0.18 g in negative control experiment (Fig. 8a, d).

On the other hand, there was no significant change in ATP-induced contraction in CYP-injected rats compared to negative control experiment (Fig. 8b). The percentage change from negative control experiment was calculated for the three antagonists and compared between normal rats and rats injected with CYP and the change in ATP-induced contraction was not significantly different in CYP-injected rats compared to normal rats (Fig. 8c).

## Discussion

CYP is one of the oxazaphosphorine alkylating agents used for the management of different types of tumors, such as myeloma and lymphoma. In addition, it has been used in the management of other diseases, such as rheumatoid arthritis (Levine and Richie 1989). Hemorrhagic cystitis is considered one of the severe side effects of CYP. According to earlier research, hemorrhagic cystitis is caused by the CYP metabolic product acrolein. When acrolein builds up in the bladder, it damages the bladder and causes the release of several inflammatory enzymes and cytokines (Ribeiro et al. 2012). In the current study, urinary bladder damage was confirmed using histopathological examination.

Furthermore, our results demonstrated that EFS, ACh, and ATP-induced contractions of detrusor muscles in rats with CYP-induced hemorrhagic cystitis were significantly diminished in line with, our previously published data (Bassiouni et al. 2019a), as well as others (Aronsson et al. 2014; Engin et al. 2021). The decrease in the contractility of isolated detrusor muscles in CYP-injected rats is probably attributed to the downregulation of both muscarinic and P2X receptors due to the increase in the cholinergic and purinergic neuronal activity during cystitis, a notion that was confirmed by the decrease in binding sites for radioligands (Kageyama et al. 2008; Ogoda et al. 2016).

Prostanoids play an important role in the control of the bladder/voiding function peripherally, but their modulatory role on cholinergic and purinergic pathways in physiological and pathological conditions needed further investigation. According to our results, alprostadil significantly amplified both EFS and ACh-induced contraction of isolated detrusor muscles in normal rats and rats with cystitis. Regarding ATP-induced contraction, the amplification induced by alprostadil was only significant in normal rats at high concentrations. Morita et al. (1994) reported that PGE_1_ increased the force of contraction of isolated rabbit urinary bladder smooth muscles, and Palea et al. (1998) reported that PGE_1_ induced weak contractions of isolated human urinary bladder smooth muscles. The current study is consistent with what was reported by Bassiouni et al. (2019a), who showed that alprostadil amplified EFS and ACh-induced contractions of isolated detrusor muscles in normal rats and in rats injected with CYP. A previous study showed that indomethacin had an antagonistic effect on ATP-induced contraction of isolated rabbits’ detrusor muscles (Dean and Downie 1978). Husted et al. (1983) also reported that PGE_2_ and PGF_2α_ counteracted the inhibition of ATP-induced phasic contractions caused by indomethacin. It has been reported that PGE_1_ binds to the EP_1-4_ receptors, and the order of its affinity for these receptors is EP_4_ > EP_3_ > EP_2_ > EP_1_ (Boie et al. 1997). As a G_q_-coupled receptor that raises intracellular Ca^2+^ concentrations, EP_1_ is implicated in contraction. G_s_-coupled receptors EP_2_ and EP_4_ are implicated in relaxation because they activate AC and raise cyclic adenosine monophosphate (cAMP). Since EP_3_ is a G_i_-coupled receptor that inhibits AC, it plays a role in contraction (Coleman et al. 1994; Hata and Breyer 2004; Norel et al. 2020). Therefore, EP_1_ and EP_3_ can be the receptors involved in amplification of contraction induced by alprostadil. The current study showed that the amplification induced by alprostadil was significantly higher in CYP-injected rats compared to normal rats regarding ACh-induced contraction, with no significant difference in the amplification of EFS or ATP-induced contraction between normal and CYP-injected rats. Previous studies reported that PGE_2_ and the expression of EP_1_ and EP_2_ receptors increase in the bladders of rats with interstitial cystitis (Zhang et al. 2016). This may explain that the interaction between the cholinergic pathway and PGE_1_ increases during cystitis through the contractile EP_1_ receptor. In contrast, Chuang et al. (2010) reported that in rats injected with CYP, EP_1_ receptor expression was downregulated while EP_4_ receptor expression was upregulated with no significant change in EP_2_ and EP_3_ receptor expression. Measurements of PGE_1_ levels and EP_1-4_ receptor expression in both normal rats and rats with cystitis are required to confirm this assumption.

Like alprostadil, selexipag had the same effect on EFS, ACh, and ATP-induced contractions in normal rats and rats with cystitis. Selexipag is a selective IP receptor agonist and Kuwano et al. (2007) showed that it had a weak affinity to other prostanoid receptors. IP receptor is a G_s_-coupled receptor leading to the activation of AC and cAMP increase; however, other studies showed that IP receptor also coupled to G_q_ protein and increased intracellular Ca^2+^ (Namba et al. 1994; Norel et al. 2020). This is consistent with a previous study that showed that PGI_2_ contracted the isolated porcine detrusor muscles, but its effect was small compared to other prostanoids used in the study (Stromberga et al. 2020). The current study showed that the amplification induced by selexipag was significantly higher in CYP-injected rats compared to normal rats regarding EFS and ACh-induced contraction, with no significant difference in the amplification of ATP-induced contraction between normal and CYP-injected rats. During inflammation, PGI_2_ production is elevated (Ricciotti and FitzGerald 2011). This may explain the increase in amplification induced by selexipag either on neuronal or on cholinergic mediated contraction, and that interaction between IP receptor and both neuronal and cholinergic pathways may increase during cystitis. Measurements of PGI_2_ levels and IP receptor expression in both normal rats and rats with cystitis are required to confirm this assumption. It has to be noted that part of the effect of alprostadil and selexipag may be mediated by other mechanisms, but this has not been tested.

We used SC51322, a specific EP_1_ receptor antagonist, and RO1138452, a selective IP receptor antagonist, to investigate the potential involvement of EP_1_ and IP receptors in EFS, ACh, or ATP-induced contractions of the isolated rats’ detrusor muscles (Abramovitz et al. 2000; Bley et al. 2006). EP_1_ receptor antagonist decreased EFS-induced contraction in both normal rats and rats with cystitis, and the decrease was significant at higher frequency with no significant difference between normal and CYP-injected rats. EP_1_ receptor antagonist also decreased ACh-induced contraction, but the decrease was significant in rats injected with CYP and at higher concentrations. IP receptor antagonist also decreased EFS and ACh-induced contraction in normal rats and rats with cystitis, and the decrease was significant at higher frequency and higher concentrations of ACh. It has to be noted that the inhibition induced by IP receptor antagonist was not significantly different between normal rats and rats injected with CYP regarding EFS-induced contraction; however, the inhibition in ACh-induced contraction was less in rats with cystitis compared to normal rats with a significance at ACh concentration (10^−4^ M). A previous study showed that nitric oxide (NO) donor at lower concentrations significantly amplified ACh-induced contractions; however, higher concentrations of NO donor significantly attenuated ACh-induced contraction (Bassiouni et al. 2019b). Another previous study showed that the expression of inducible nitric oxide synthase (iNOS) was increased in rat bladder smooth muscle cell culture after the exposure to plasma from rats treated with CYP (Xu et al. 2001). Thus, the increase in NO production during cystitis may have a role in the inhibition of ACh-induced contraction, masking part of the inhibitory effect of IP receptor antagonist. However, this assumption needs further investigation.

Regarding ATP-induced contraction, the effect of EP_1_ and IP receptor antagonists was investigated and compared to the negative control experiment. It was observed that both antagonists reduced the ATP-induced contraction, and the inhibitory effect was significant in normal rats and at high concentrations of ATP with no significant change in rats with cystitis. This suggests that the interaction between the purinergic pathway and both EP_1_ and IP receptors occurs in normal physiological condition rather than in hemorrhagic cystitis.

Previous studies showed that TP receptor agonist induced the contraction of isolated human detrusor muscles, and that effect was antagonized in the presence of TP receptor antagonist (Palea et al. 1998). Another study showed that TP receptor agonist at high concentrations induced small contractions in isolated rat detrusor muscles (Root et al. 2015). TP receptors were shown to be G_q_ coupled and to raise intracellular Ca^2+^. They were also found to be G_12/13_ coupled, which activated the Rho signaling cascade and caused smooth muscle contraction. Others reported that TP receptors coupled to G_i_ and G_s_ proteins (Woodward et al. 2011). The role of TP receptor in neuronal-, cholinergic-, and purinergic-mediated detrusor muscle contractions was investigated in the current study using S18886, potent TP receptor antagonist (Cimetière et al. 1998). The current study showed that TP receptor antagonist had no significant effect on neuronal or cholinergic mediated contraction of isolated detrusor muscles in normal rats and in case of cystitis, suggesting that there is no interaction between TP receptor and these pathways. On the other hand, TP receptor antagonist inhibited ATP-induced contraction in normal rats, and the inhibition was significant compared to the negative control experiment at high concentrations of ATP with no significant change in case of rats with cystitis. This can be explained that TP receptors interact with the purinergic pathway in normal physiological condition but during cystitis, TP receptors may be downregulated. However, more studies are required to measure TP receptors expression to confirm this hypothesis.

## Conclusion

Urinary bladder damage in CYP-induced hemorrhagic cystitis was histopathologically confirmed. CYP-induced hemorrhagic cystitis was also associated with a decrease in both neurogenic contractility of detrusor muscle and direct contractility using ACh or ATP. Alprostadil may play an important role in the increase of neurogenic contractility, by maybe endogenous ACh or ATP, as well as ACh-induced contractility in both normal condition and cystitis; the highest amplifying effect was observed on ACh-induced contraction in the case of cystitis. Selexipag shows potential of increasing the neurogenic contractility and ACh-induced contractility of normal bladder detrusor muscle and to a greater extent in cystitis. For this reason, both selexipag and alprostadil may have a potential beneficial action in hemorrhagic cystitis and merit further investigation. Both alprostadil and selexipag at higher concentrations amplified the purinergic signaling but only in normal physiological condition.

EP_1_ receptors seem to have a role in neurogenic detrusor muscle contractility in normal physiological condition and in cystitis. In addition, it seems to play an important role in ACh-induced contractility in case of cystitis. IP receptors also seem to have a role in both neurogenic detrusor contractility and ACh-induced contractility in normal condition and cystitis. EP_1_, IP, and TP receptors seem to be involved in purinergic signaling in normal physiological condition.

Measurement of prostanoid levels and prostanoid receptor expression in both normal condition and cystitis remains essential to confirm the results of the current study.

## Data Availability

All source data for this work (or generated in this study) are available upon reasonable request.
